# Esomeprazole Increases Airway Surface Liquid pH in Primary Cystic Fibrosis Epithelial Cells

**DOI:** 10.3389/fphar.2018.01462

**Published:** 2018-12-11

**Authors:** Livia Delpiano, Joseph J. Thomas, Annabel R. Yates, Sarah J. Rice, Michael A. Gray, Vinciane Saint-Criq

**Affiliations:** ^1^Epithelial Research Group, Institute for Cell and Molecular Biosciences, Faculty of Medical Sciences, Newcastle University, Newcastle upon Tyne, United Kingdom; ^2^Skeletal Research Group, Institute of Genetic Medicine, International Centre for Life, Newcastle University, Newcastle upon Tyne, United Kingdom

**Keywords:** airway surface liquid pH, cystic fibrosis, ATP12A, ouabain, proton pump inhibitor, esomeprazole

## Abstract

Respiratory failure, driven by airways mucus obstruction, chronic inflammation and bacterial infections, is the main cause of mortality and morbidity in people with cystic fibrosis (CF) due to defects in the Cl^-^ and ${\text{HCO}}_{3}^{-}$ transport activity of the CF Transmembrane conductance Regulator (CFTR). Most recent pre-clinical and clinical studies have focused on restoring CFTR function by enhancing its trafficking or transport activity and show promising results. However, there are a significant number of patients that will not benefit from these CFTR-targeted therapies and it is therefore important to identify new non-CFTR targets that will restore lung function, by-passing CFTR dysfunction. The H^+^/K^+^-ATPase, ATP12A, has recently been identified as a potential novel target for CF therapies, since its acute inhibition by ouabain was shown to help restore mucus viscosity, mucociliary transport, and antimicrobial activity using *in vitro* CF airway models, and this effect was linked to an increase in the pH of the airway surface liquid (ASL). Here, we have evaluated the potential therapeutic use of ouabain by investigating the effect of chronically treating fully differentiated CF primary human airway epithelial cells (hAECs) with ouabain, under thin film conditions, resembling the *in vivo* situation. Our results show that although chronic treatment increased ASL pH, this correlated with a deleterious effect on epithelial integrity as assessed by LDH release, transepithelial electrical resistance, fluorescein flux, and ion transport. Since ATP12A shares approximately 65% identity with the gastric H^+^/K^+^-ATPase (ATP4A), we investigated the potential of using clinically approved ATP4A proton pump inhibitors (PPIs) for their ability to restore ASL pH in CF hAECs. We show that, despite not expressing ATP4A transcripts, acute exposure to the PPI esomeprezole, produced changes in intracellular pH that were consistent with the inhibition of H^+^ secretion, but this response was independent of ATP12A. More importantly, chronic exposure of CF hAECs to esomeprazole alkalinized the ASL without disrupting the epithelial barrier integrity, but this increase in ASL pH was consistent with a decrease in mRNA expression of *ATP12A*. We conclude that PPIs may offer a new approach to restore ASL pH in CF airways, which is independent of CFTR.

## Introduction

Cystic fibrosis (CF) is the most common autosomal recessive genetic disease in Caucasian populations and affects more than 70,000 people worldwide (Kelly, 2017). It is due to mutations in the Cystic Fibrosis Transmembrane conductance Regulator (*CFTR*) gene encoding an anion channel, transporting primarily Cl^-^ and ${\text{HCO}}_{3}^{-}$ in epithelial tissues (Saint-Criq and Gray, 2017). CF is characterized by severe lung pathophysiology where thick, sticky, mucus provides a favorable environment for bacterial colonization, which, together with the initial CFTR defect, are the cause of a chronic inflammation that leads ultimately to organ failure. The CFTR channel is an essential regulator of the airway surface liquid (ASL) composition (Namkung et al., 2009; Van Goor et al., 2009; Luan et al., 2017). This thin fluid layer lines the airway epithelium, and contributes to the efficient physical and chemical barrier mechanism against inhaled particles and pathogens by regulating ciliary beating, mucociliary transport, and antimicrobial activity. Through its Cl^-^ and ${\text{HCO}}_{3}^{-}$ transport activities, CFTR controls water movement across the epithelium and thus ASL hydration as well as its pH, respectively. However, the absolute value of the ASL pH in people with CF is still controversial as the measurement of this parameter in such a thin layer of fluid has proven very difficult. Although previous reports have shown an acidic ASL pH in human and animal models of CF airways (Coakley et al., 2003; Song et al., 2006; Pezzulo et al., 2012; Birket et al., 2018), the most recently published *in vivo* study reported no difference in ASL pH between children with or without CF (Schultz et al., 2017). Nevertheless, multiple studies have shown the importance of pH homeostasis in the ability of the airways to maintain ASL hydration (Garland et al., 2013), fight infections (Berkebile and McCray, 2014; Tang et al., 2016) and remove trapped microorganisms from the lungs (Quinton, 2008; Tang et al., 2016). Therefore increasing ${\text{HCO}}_{3}^{-}$ or inhibiting H^+^ secretion could be a suitable therapeutic strategy for lung disease in CF. To date, most pre-clinical research has focused on restoring CFTR function using CFTR-directed therapeutics. Gating mutants such as G551D (and others) respond very well to the CFTR potentiator, Ivacaftor, as well as a number of residual function mutations (De Boeck and Amaral, 2016) and next generation correctors appear able to restore some function to the most common CF-causing mutation (F508del) (Taylor-Cousar et al., 2017; Vertex, 2017). However, around 15% of people with CF lack the F508del mutation in both alleles and a certain percentage of these individuals who express F508del in at least one allele, experienced limited benefit from next generation CFTR modulators (NCT01225211; Boyle et al., 2014; Rowe et al., 2017). Therefore, there is an unmet need for alternative, mutation-independent, therapies that restore lung function in all people with CF. Accordingly, targeting non-CFTR H^+^ or ${\text{HCO}}_{3}^{-}$ channels or transporters, is a promising therapeutic strategy.

A recent study in mouse, pig and human airways has shown the essential role of the non-gastric H^+^/K^+^-ATPase, ATP12A, in ASL pH regulation in CF (Shah et al., 2016). Here the absence of expression of this ATPase in mice was linked to the mild pulmonary phenotype in the CF animals. On the other hand, acute (2 h) inhibition of this pump in pig and human airway cultures by a high concentration of apical ouabain, increased ASL pH and restored bacterial killing and mucus viscosity. This study showed for the first time the potential therapeutic use of ouabain in CF airways by targeting ASL pH homeostasis. However, to our knowledge no one has investigated the long-term effects of apical exposure to this widely used cardiac glycoside, that also inhibits the basolateral Na^+^/K^+^-ATPase, a key transporter essential for transepithelial ion and fluid transport, on airway epithelial cells. Moreover, as ATP12A belongs to the family of hydrogen-potassium ATPases (Crambert et al., 2002) and shares significant sequence and functional homology with the gastric form of the H^+^/K^+^-ATPase (ATP4A), for which there are a number of well-characterized proton pump inhibitors (PPIs) (Iwakiri et al., 2016) in use clinically, it was of interest to test the efficiency of these PPIs on airway cell function.

Therefore, in this study we tested the effects of long-term apical ouabain treatment on CF primary human airway epithelial cells (hAECs) ASL pH and epithelial integrity and compared this to the response to one of the commonly used PPIs, esomeprazole. We show for the first time that although apical treatment with ouabain increased ASL pH in a dose-dependent manner, this was positively correlated with an increase in cytotoxicity and disruption of epithelial barrier function. On the other hand, even though the gastric H^+^/K^+^-ATPase was not expressed in airway epithelial cells, exposure to esomeprazole acidified the cytosol and increased ASL pH of primary CF hAECs. We show that esomeprazole had a dual mechanism of action: acutely, it induced intracellular acidification in an ATP12A-independent manner but, chronic exposure, which importantly did not have any deleterious effect on epithelial integrity, was linked to decreased *ATP12A* mRNA levels. These results open up the possibility of repurposing PPIs as a new therapeutic approach for treating CF lung disease.

## Materials and Methods

### Chemicals

Ouabain (O3125), esomeprazole (E7906), *N*-Acetyl-L-cysteine (A7250), amiloride (A7410), fluorescein (F6377), mitomycin C (M0503), and UTP (U6750) were purchased from Sigma-Aldrich. Forskolin (1099), Y-27632 (1254), and CFTRInh172 (3430) were purchased from Tocris (RnD). The fluorescent dyes, Alexa Fluor^TM^ 488- Dextran (D22910); pHrodo^TM^ Red Dextran (P10361) and BCECF, AM (2′,7′-*Bis*-(2-Carboxyethyl)-5-(and-6)-Carboxyfluorescein, Acetoxymethyl Ester) (B1150) were purchased from ThermoFisher Scientific.

### Solutions

See Table 1.

**Table 1:** Composition of the solutions used in this study. Concentrations are given in mM.

	${\text{HCO}}_{3}^{-}$ KRB	low Cl^-^	NaCl HEPES	High K^+^ HEPES/Nigericin	K^+^-free solution (0K^+^)	ASL pH standard curve solution	ASL pH standard curve solution	ASL pH standard curve solution
*pH at 37°C*	*7.4*	*7.4*	*7.4*	*6/6.9/7.5*	*7.4*	*5.5-6-6.5*	*7-7.5*	*8*
NaHCO_3_	25	25			25			
NaCl	115		130	5	120	86	86	86
KCl	5	5	5	130		5	5	5
CaCl_2_	1	1.2	1	1	1	1.2	1.2	1.2
MgCl_2_	1	1.2	1	1	1	1.2	1.2	1.2
D-Glucose	5	5	5	5	5			
Na-gluconate		115						
K_2_SO_4_								
Ca-gluconate		2.8						
Mg-gluconate								
NaHEPES			10	10			100	
MES						100		
Tris								100

### Cell Culture

Primary non-CF (*n* = 3 donors) and CF (*n* = 3 donors, all F580del/F508del) hAECs were a kind gift from Dr. Scott H. Randell (Marsico Lung Institute, The University of North Carolina at Chapel Hill, United States). The cells were obtained under protocol #03-1396 approved by the University of North Carolina at Chapel Hill Biomedical Institutional Review Board. Primary cells from three different CF donors (all F580del/F508del) were obtained via the CFFT Biorepository. They were expanded using the conditionally reprogrammed cell (CRC) culture method as previously described (Suprynowicz et al., 2012). Briefly, cells were seeded on 3T3J2 fibroblasts inactivated with mitomycin C (4 μg/ml, 2 h, 37°C) and grown in medium containing the ROCK inhibitor Y-27632 (10 μM) until they reached 80% confluence. Cells then underwent double trypsinization to remove the fibroblasts first and then detach the hAECs from the P150 dish. At that stage, cells were counted and could be frozen down. Cryopreserved cells were seeded onto semi-permeable supports (6.5 or 12 mm) in bilateral differentiating medium (ALI medium) as previously described (Randell et al., 2011). The apical medium was removed after 3–4 days and cells then allowed to differentiate under air-liquid interface (ALI) conditions. Ciliogenesis started approximately 12–15 days after seeding and cells were used for experiments between days 25 and 35 after seeding.

### Knock-Down of ATP12A Using CRISPR-Cas9

Guide RNA (gRNA) sequences targeting upstream (5′-GGCCGGAGGGAGTCGGACAG-3′) and downstream (5′-TCCCTCAGACTGAATGTCTG-3′) of *ATP12A* exon 2 were designed using the Optimized CRISPR Design Tool^1^. Single stranded DNA oligonucleotides (IDT) containing the gRNA sequence in addition to the *BbsI* restriction enzyme sequences were annealed (95 to 25°C, Δ-6°C/min). The double stranded oligonucleotides were then ligated into the CRISPR-Cas9 vector, PX462, following *BbsI* linearization, using T4 ligase (Invitrogen) overnight at 16°C. To validate the sequences, cells from the human chondrocyte cell line Tc28a2 were nucleofected [10^6^ cells, 5 μg of plasmid DNA using the manufacturer recommended Cell Line 4D-Nucleofector X Kit in combination with the 4D-Nucleofector System (Lonza)] and selected with puromycin after 24 h. Following expansion, nucleic acids were extracted using the EZNA DNA/RNA Isolation kit (Omega Bio-Tek) according to the manufacturer’s protocol. Deletion of the target region was confirmed using end-point PCR (Supplementary Figure 6D).

Following validation in the Tc28a2 cells, primary CF hAECs were transfected using an adapted version of a previously published protocol (Ramachandran et al., 2013). Briefly, DNA (3 μg) was incubated for 3 min in ALI medium without antibiotics, FuGENE HD Transfection Reagent was then added (3:1 ratio) and further incubated for 15 min at room temperature on the collagen-coated semi permeable supports. Freshly thawed cells were counted and seeded onto the semi-permeable support in antibiotics-free ALI medium. After 5 h incubation, medium was replaced and cells were grown as previously described in antibiotics containing ALI medium from day 3. After pH_i_ or ASL pH experiments, RNA was extracted as described below and *ATP12A* mRNA was quantified by RT-qPCR.

### ASL pH Measurements

Cells grown on 6.5 mm transwells were washed apically with 120 μl glucose-free ${\text{HCO}}_{3}^{-}$-containing Krebs solution (${\text{HCO}}_{3}^{-}$ KRB, Table 1) for 15 min at 37°C, 5% CO_2_. The ASL was stained using 3 μl of a mixture of dextran-coupled pH-sensitive pHrodo Red (0.5 mg/ml, λex: 565 nm, λem: 585 nm) and Alexa Fluor^®^ 488 (0.5 mg/ml, λex: 495 nm, λem: 519 nm) diluted in glucose-free ${\text{HCO}}_{3}^{-}$ KRB, overnight at 37°C, 5% CO_2_. Alexa Fluor^®^ 488 was used as a loading control as pHrodo is not a ratiometric dye. The next day, fluorescence was recorded using a temperature and CO_2_-controlled plate reader (TECAN SPARK 10M). After subtracting background values from pHrodo and Alexa Fluor^®^ 488, ratios were generated for each time point and pH was calculated from a standard curve where pH was clamped using highly buffered solutions between 5.5 and 8 (see Table 1 for composition). To prevent inter-experiment variability, the standard curve calibration was performed on each independent experiment. For ASL pH experiments involving apical exposure to chemicals, cells were treated overnight with the compounds, which were added with the fluorescent dyes at 0.1X final concentration. The final concentrations of the drugs added was calculated assuming a theoretical final ASL volume of 0.3 μl (10 μm × π × 3.25 mm^2^), after absorption of the excess fluid by the epithelium. ASL pH measurements were performed in duplicate.

### Transepithelial Electrical Resistance, Fluorescein Flux, and Short-Circuit Current Measurements in Ussing Chamber

Cells grown on 6.5 mm inserts were mounted into the EasyMount Ussing Chamber Systems (VCC MC8 Physiologic Instrument) and bathed in basolateral ${\text{HCO}}_{3}^{-}$ KRB and apical low Cl^-^ (Table 1) continuously gassed and stirred with 5% (v/v) CO_2_/95% (v/v) O_2_ and maintained at 37°C. Monolayers were voltage-clamped to 0 mV and monitored for changes in short-circuit current (ΔIsc) using Ag/AgCl reference electrodes. The transepithelial short-circuit current (Isc) and the TransEpithelial Electrical Resistance (TEER) were recorded using Ag–AgCl electrodes in 3 M KCl agar bridges, as previously described (Saint-Criq et al., 2013), and the Acquire & Analyze software (Physiologic Instruments) used to perform the analysis.

### Fluorescein Flux

A 100 μl sample (blank) was taken from the apical bath 5 min after mounting the monolayers and 100 μl of fluorescein (20 μg ml^-1^ final concentration) was then added to the basolateral bath. Apical samples (100 μl) were collected every 5 min for 45 min and replaced by the same volume of fresh warmed low Cl^-^ solution. Collected samples were loaded onto a 96 well plate and fluorescence was measured using a plate reader (λex: 460 nm; λem: 515 nm). Data is presented as the slope of fluorescein appearance in the apical bath and was obtained after plotting (sample-blank) vs. time and performing a linear regression. The addition of the fluorescein solution, as well as the collection and replacement of apical samples, did not have any effect on the TEER or Isc.

### Short-Circuit Current Measurements

After the last fluorescein flux samples were collected, cells were left to equilibrate for a further 10 min and ion transport agonists and inhibitors were added following this sequence: amiloride (10 μM, apical), Forskolin (Fsk, 10 μM, bilateral), CFTRinh172 (172, 20 μM, apical) and UTP (100 μM, apical). Results were normalized to an area of 1 cm^2^ and expressed as Isc (μAmp.cm^-2^).

### Intracellular pH Measurements

Primary airway epithelial cells were grown on 12 mm Transwell inserts and loaded with the pH-sensitive, fluorescent dye BCECF-AM (10 μM) for 1 h in a Na-HEPES buffered solution (see Table 1) at 37°C. Cells were mounted on to the stage of a Nikon fluor inverted microscope and perfused with a modified Krebs solution gassed with 5% (v/v) CO_2_/95% (v/v) O_2_. Solutions were perfused across the apical and basolateral membranes at 37°C at a speed of 3 and 6 ml min^-1^, respectively. Intracellular pH (pH_i_) was measured using a Life Sciences Microfluorimeter System in which cells were alternately excited at 490 and 440 nm wavelengths every 1.024 s with emitted light collected at 510 nm. The ratio of 490 to 440 nm emission was recorded using PhoCal 1.6 b software and calibrated to pH_i_ using the high K^+^/nigericin technique (Turner et al., 2016) in which cells were exposed to high K^+^ solutions containing 10 μM nigericin, set to a desired pH, ranging from 6 to 7.5. For analysis of pH_i_ measurements, ΔpH_i_ was determined by calculating the mean pH_i_ over 60 s resulting from treatment. The initial rate of pH_i_ change (ΔpH_i_/Δ*t*) was determined by performing a linear regression over a period of at least 40 s.

### RNA Extraction and Real-Time Quantitative PCR Analysis

RNA isolation from cells was performed using PureLink^®^ RNA Mini Kit (12183018A, Ambion, Life Technologies), following the manufacturer’s instructions. Briefly, lysates were mixed with 70% ethanol and loaded onto a silica-membrane column. Columns were washed with different buffers and total RNA was eluted in DNAse and RNAse-free water and stored at -80°C until use. DNase treatment was performed on 300 ng RNA prior to Reverse Transcription Polymerase Chain Reaction (RT-PCR) using RNAse-free DNAse I (04716728001, Roche) at 37°C for 10 min. Reaction was then stopped by increasing the temperature to 70°C for 10 min. Complementary DNA (cDNA) was synthesized from total RNA (300 ng) using M-MLV Reverse Transcriptase (Promega) as per supplier’s protocol (1 h at 37°C followed by 10 min at 70°C).

Real-time quantitative PCR (qPCR) was performed in a total volume of 15 μl using 2× LightCycler^®^ 480 SYBR Green I Master (Roche, 04707516001), 1.5 μl of cDNA, 2 μM forward primer and 2 μM reverse primer in a 96-well plate. Primer sequences are shown in Table 2. The expression of GAPDH was used as internal control. PCR was run with the standard program: 95°C 10 min, 40 times of cycling 95°C 15 s and 60°C 1 min in a 96-well plate. Results are shown as Threshold Cycle (Ct) or Relative quantity of mRNA copies to the determined control condition. A Ct value of 40 was applied for samples in which no messenger was detected.

**Table 2:** qRT-PCR primers for mRNA quantification.

Target	Oligo sequence	PCR product length
*ATP12A*	5′-GGGGCACACTTGTTCATCTTCTGA-3′	128
	5′-GCAAAACATCAGTGAGCATCCTG-3′	
*ATP4B*	5′-GGCCTTCTACGTGGTGATGAC-3′	136
	5′-CCCGTAAACATCCGGCCTTA-3′	
*ATP4A*	5′-AAGATCTGCAGGACAGCTACGG-3′	200
	5′-CTGGAACACGATGGCGATCA-3′	
*GAPDH*	5′-TGC ACC ACC AAC TGC TTA GC-3′	87
	5′-GGC ATG GAC TGT GGT CAT GAG-3′	

### Cell Cytotoxicity Measurement

Cell cytotoxicity was measured by quantification of secreted lactate dehydrogenase (LDH), after overnight exposure to vehicle or drugs. This was calculated after measurement of the apical + basolateral LDH release (LDH*_sec_*) and expressed as a ratio to the cell lysates LDH (PBS/Triton X-100 0.8%; LDH*_lysate_*). Samples were diluted in PBS/Triton X-100 0.8%, loaded onto a 96 well plate (Costar) and incubated with the assay reagent for 30 min at room temperature in the dark. After addition of the stop solution, absorbance was measured at 490 nm (kit CytoTox96^®^ Non-Radioactive Cytotoxicity Assay kit, Promega) in a TECAN Infinite M200pro plate reader. Data were analyzed and cytotoxicity was calculated as follows:

$$\text{\% cytotoxicity}=\left(\frac{{\text{OD LDH}}_{\text{sec}}}{{\text{OD LDH}}_{\text{sec}}+{\text{OD LDH}}_{lysate}}\right)\times 100$$

### Statistical Analysis

All analyses were performed using GraphPad Prism7. Where applicable, results are shown as mean ± SEM. Dose responses were analyzed by non-linear regression on a log scale (log[drug] vs. response) and IC_50_ were compared by the extra sum-of-squares *F*-test. For some data, correlations were tested and Pearson correlation coefficient given in the text. Parametric and non-parametric data distributions were assessed with the D’Agostino & Pearson normality test. Multiple group and two-group comparisons were performed using appropriate statistical tests for specific data sets (see details in individual figure legends).

## Results

### The Activity of ATP12A Is Not Different Between CF and Non-CF hAECs

**FIGURE 1:**
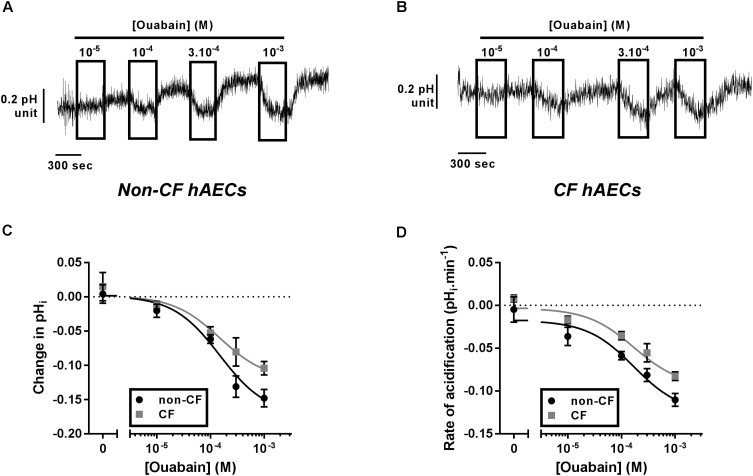
Characterization of ATP12A activity in non-CF and CF primary hAECs. Non-CF and CF hAECS acidify in response to acute apical ouabain. Upper panels show representative traces of pH_i_ responses to acute increasing apical concentrations of ouabain in non-CF **(A)** and CF **(B)** hAECs. Change in pH_i_ **(C)** and rate of acidification **(D)** of non-CF (black circles, *n* ≥ 7, three donors) and CF (gray squares, *n* ≥ 5, five donors) hAECs in response to acute increasing concentrations of apical ouabain. Results show no significant difference in change in pH_i_ or rate of acidification between non-CF and CF hAECs (non-linear regression with comparison of fits; change in pH_i_: *p* = 0.51; rate: *p* = 0.64).

As ATP12A was suggested to be a key regulator of ASL pH (Shah et al., 2016), we investigated its expression and activity in fully differentiated primary cultures of CF and non-CF primary hAECs. Quantitative (q)PCR showed that the level of mRNA expression of *ATP12A* was not different between primary CF and non-CF hAECs (Supplementary Figure 1). Because ATP12A has previously been shown to be constitutively active in airway cells (Coakley et al., 2003; Shah et al., 2016; Lennox et al., 2018), inhibition of this pump, by acute exposure to ouabain, would be predicted to cause an intracellular acidification. Therefore, to functionally assess ATP12A activity, we measured the acute effect of increasing concentrations of apical ouabain on intracellular pH (pH_i_) in primary CF and non-CF hAECs, loaded with the pH-sensitive dye BCECF-AM (see section “Materials and Methods”). Figures 1A,B shows that, as predicted, acute exposure to apical ouabain lead to a dose-dependent, reversible, acidification in both non-CF and CF cultures, respectively. Analysis of the dose-response curves showed that although the maximal change in pH_i_ induced by 1 mM ouabain was somewhat lower in CF versus non-CF cells, the IC_50_ calculated from the change in pH_i_ (Figure 1C) as well as from the rate of acidification (Figure 1D) induced by ouabain were not significantly different (IC_50_: ΔpH_i_ non-CF = 165.2 μM, *n* ≥ 7; CF = 102.3 μM, *n* ≥ 5, *p* = 0.506; rates of acidification non-CF = 153.6 μM, *n* ≥ 7; CF = 217.4 μM, *n* ≥ 5, *p* = 0.636). Our results, obtained from 12 independent experiments (*n* = 7 non-CF/2 different donors and *n* = 5 CF/3 different donors), also showed that baseline pH_i_ was not significantly different between non-CF and CF hAECs. Due to the potential beneficial effect of inhibiting H^+^ secretion in CF epithelia, the rest of the study then focused on primary CF hAECs.

### Chronic Apical Ouabain Treatment Alkalinized the ASL

**FIGURE 2:**
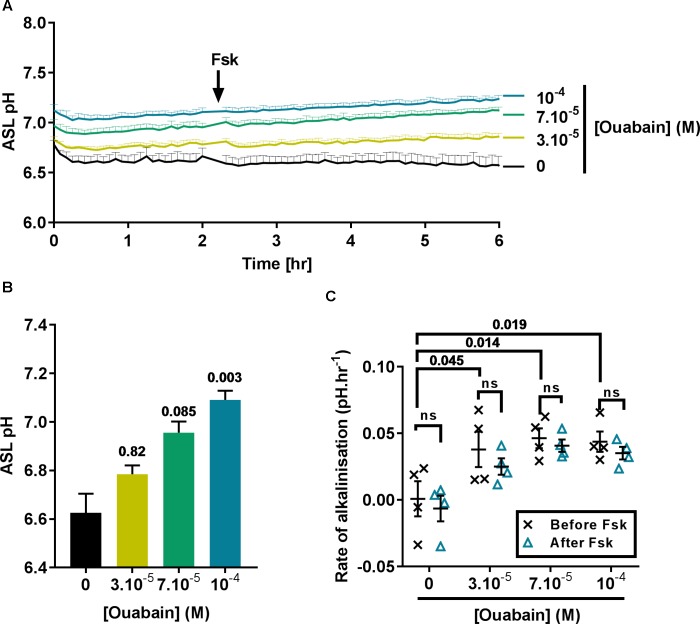
Ouabain increases ASL pH in primary CF hAECs in a dose-dependent manner. CF hAECs were treated overnight with increasing concentrations of apical ouabain and ASL pH was then measured in real time **(A)** under resting conditions and after stimulation with forskolin (Fsk, basolateral, 10 μM) (*n* = 4 in duplicate, three donors; data plotted as mean ± SEM). **(B)** Chronic ouabain increased resting ASL pH. Resting ASL pH was calculated as an average of five points before addition of Fsk (Friedman’s test comparing ouabain-treated vs. vehicle-treated). **(C)** CF hAECs treated with ouabain show a slow and continuous increase in ASL pH but do not respond to Fsk. The rate of increase in pH was calculated before and after addition of basolateral Fsk and compared using a two-way repeated measures (RM)-ANOVA with Tukey’s test.

In view of the fact that acute treatment using 100 μM ouabain induced a significant intracellular acidification near to the IC_50_, we then investigated the effect of treating CF cells overnight with 30 to 100 μM ouabain on ASL pH. After overnight incubation, the cells were transferred to a CO_2_ and temperature-controlled plate-reader and ASL pH was recorded every 5 min for 2 h before addition of the cAMP agonist forskolin (Fsk) to the basolateral compartment, and ASL pH recorded for a further 4 h. In the vehicle (DMSO)-treated cells, ASL pH was stable over the entire course of the experiment (Figure 2A, black line) whereas, ouabain-treated cell cultures showed a slow but steady increase in ASL pH over time (Figure 2A, yellow, green, and blue lines). Although increasing concentrations of ouabain appeared to increase ASL pH in a dose-dependent manner it only reached significance with the highest dose tested, 100 μM (Figures 2A,B). Surprisingly, and contrary to what other groups have reported (Coakley et al., 2003; Shah et al., 2016) Fsk treatment did not induce ASL acidification in CF cells. As ATP12A is inhibited by ouabain or K^+^-free (0K^+^) solution, we confirmed our results using a different technique. Here, we measured changes in pH_i_ in response to ouabain and 0K^+^ in the absence or presence of 10 μM Fsk. Results showed that Fsk itself, (1) did not alter pH_i_ (Supplementary Figure 2A) and (2) did not change the extent or rate of ouabain and 0K^+^-induced acidification (Supplementary Figures 2A–C).

As ASL pH slowly increased after addition of Fsk, we determined if ouabain induced changes in CF hAECs that would have sensitized them to Fsk (thereby inducing an increase in ASL pH via CFTR), by measuring the rates of alkalinization before and after addition of Fsk. As shown in Figure 2C, the initial rates of alkalinization increased significantly in ouabain treated samples when compared to vehicle-treated cells, but these rates were unchanged after Fsk treatment, suggesting that chronic apical ouabain treatment induced changes that allowed for a slow and gradual increase in ASL pH. We hypothesized that chronic apical ouabain treatment was having a deleterious effect on CF hAECs and therefore assessed the effect of these treatments on epithelial integrity.

### Chronic Apical Ouabain Treatment Disrupts Epithelial Integrity by Inhibiting the Basolateral Na^+^/K^+^-ATPase

**FIGURE 3:**
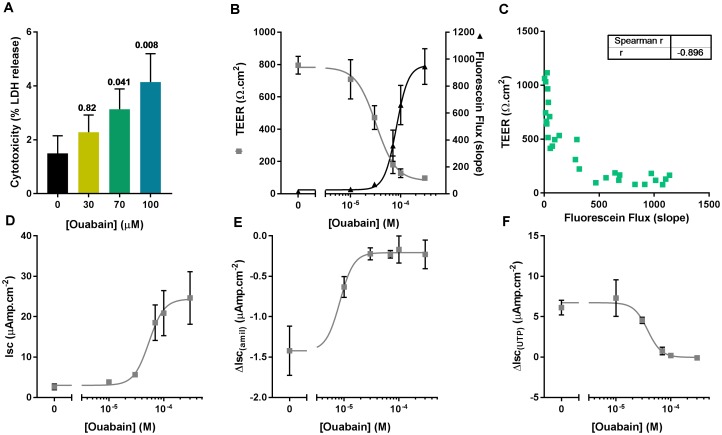
Apical ouabain disrupts epithelial integrity of primary CF hAECs. **(A)** LDH release in response to increasing concentrations of apical ouabain in primary CF hAECs (*n* = 4, three donors; Friedman’s test comparing ouabain-treated vs. vehicle-treated). **(B)** Effect of increasing concentrations of apical ouabain on TransEpithelial Electrical Resistance (TEER) and Fluorescein Flux assessed in Ussing chambers in the presence of a basolateral to apical Cl^-^ gradient (*n* = 4). **(C)** Correlation between TEER and Fluorescein Flux (Spearman correlation test: *p* < 0.001). Effect of increasing concentrations of apical ouabain on resting short-circuit current (Isc, **D**, *n* = 4), amiloride-sensitive Isc (ΔIsc_(amil)_, **E**, *n* = 4) and UTP-induced Isc (ΔIsc_(UTP)_, **F**, *n* = 4).

We first assessed the potential cytotoxic effect of the same concentrations of ouabain used in the ASL pH experiment by measuring LDH release after overnight exposure to the drug. To do this, the apical surface of CF hAECs was washed with 50 μl of ${\text{HCO}}_{3}^{-}$ KRB for 30 min (37°C, 5% CO_2_) and LDH was measured in the apical washes as well as the basolateral media. Figure 3A shows that 70 and 100 μM ouabain induced a significant increase in LDH release. Even though the percentage released remained under 10%, this increase in LDH release was positively correlated with the increase in ASL pH (Pearson *r* = 0.5278; *p* = 0.036). To confirm the deleterious effect of chronic apical ouabain treatment on epithelial integrity, we measured TEER and fluorescein flux across the epithelia as well as the activity of the main ion channels expressed in primary CF hAECs. Increasing concentrations of the ATP12A inhibitor significantly decreased TEER and increased fluorescein flux across the epithelia (Figure 3B). These two parameters were inversely correlated as shown in Figure 3C (Pearson *R* = -0.809, *p* < 0.001). Resting short circuit current increased with increasing concentrations of ouabain (Figure 3D), whereas amiloride-sensitive and UTP-induced currents were inhibited in a dose dependent manner (Figures 3E,F, respectively). Fsk-induced and CFTRinh172-sensitive Isc were minimal and remained unchanged after treatment with increasing concentrations of apical ouabain (data not shown).

**FIGURE 4:**
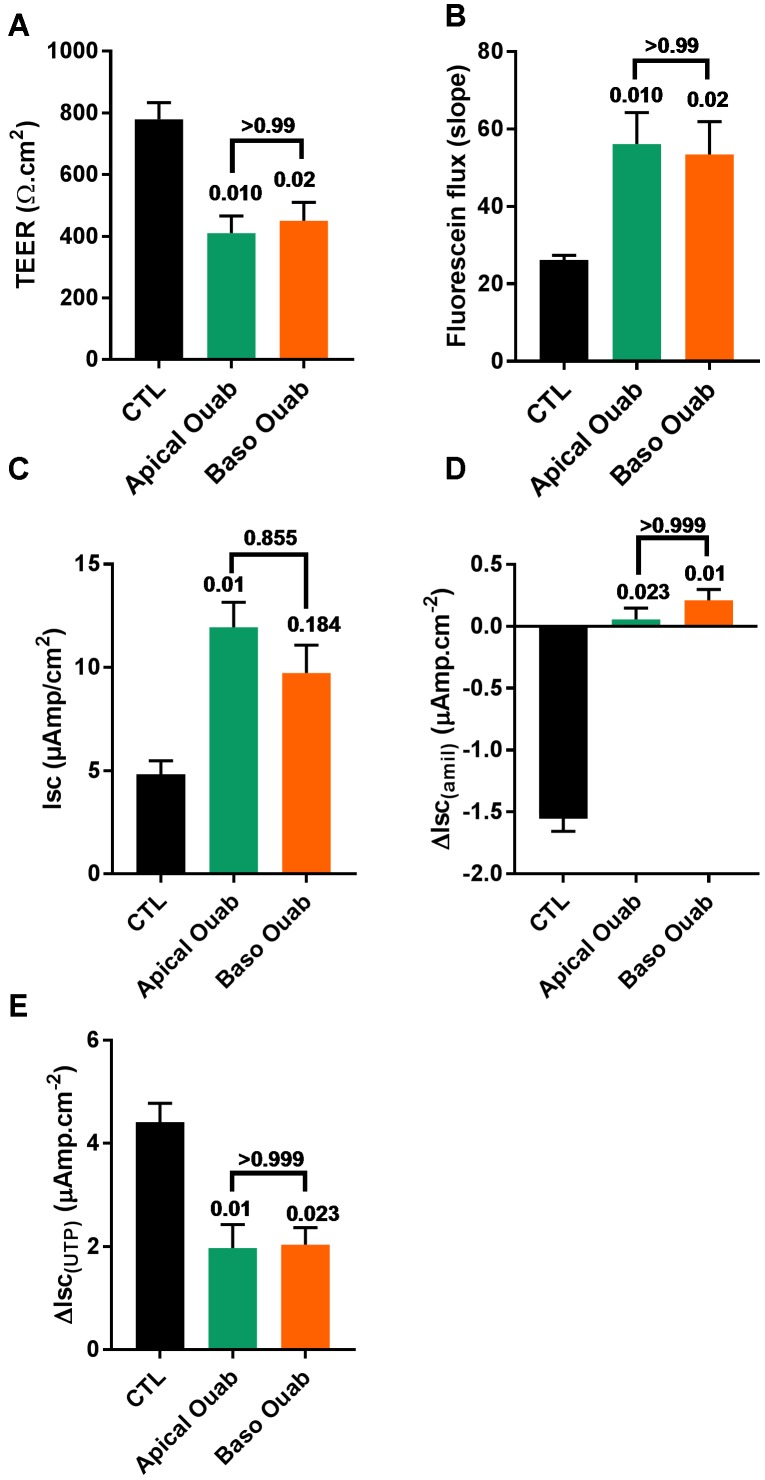
Ouabain (Ouab)-induced epithelial disruption is mimicked by inhibition of the basolateral Na^+^/K^+^-ATPase in primary CF hAECs. The effect of 50 μM apical ouabain was compared to 30 nM basolateral ouabain on TEER **(A)**, fluorescein flux **(B)**, resting Isc **(C)**, ΔIsc_(amil)_ **(D)**, and ΔIsc_(UTP)_ **(E)** (all, *n* = 7, five donors, Friedman’s test comparing all conditions).

To investigate if the deleterious effects of apical ouabain treatment were due to an inhibition of the basolateral Na^+^/K^+^-ATPase, cells were treated overnight with either 70 μM apical ouabain or the equivalent basolateral concentration (30 nM), assuming complete equilibration of the inhibitor from the apical to the basolateral compartment (70 μM × 0.3 μl/700 μl). As shown in Figure 4, treatment of CF hAECs with 30 nM basolateral ouabain induced a decrease in TEER (Figure 4A), an increase in fluorescein flux (Figure 4B) and resting Isc (Figure 4C) as well as an inhibition of amiloride-sensitive (Figure 4D) and UTP-induced (Figure 4E) changes in Isc, that were not significantly different from the changes induced by the overnight apical treatment with 70 μM ouabain. Taken together, these results show that the potential use of ouabain to target ASL pH in CF is very likely to be deleterious and cytotoxic to airway epithelial cells. To further investigate this, CF hAECs were treated basolaterally with conditioned medium derived from the basolateral compartment from cells treated apically with 30 μM ouabain (for 24 h). This revealed that conditioned medium did not fully reproduce the effects seen with apical exposure alone, which suggests that either ouabain is metabolized during the 48 h incubation or that the cytotoxic effect of ouabain cannot be solely explained by the inhibition of the serosal Na^+/^K^+^-ATPase (Supplementary Figures 3A–E). It was thus of interest to identify molecules that could specifically target apical H^+^ secretion, without affecting the Na^+^/K^+^-ATPase, as a non-cytotoxic strategy to increase ASL pH in CF airways.

### Esomeprazole Affects Intracellular pH and Raises ASL pH in CF hAECs

**FIGURE 5:**
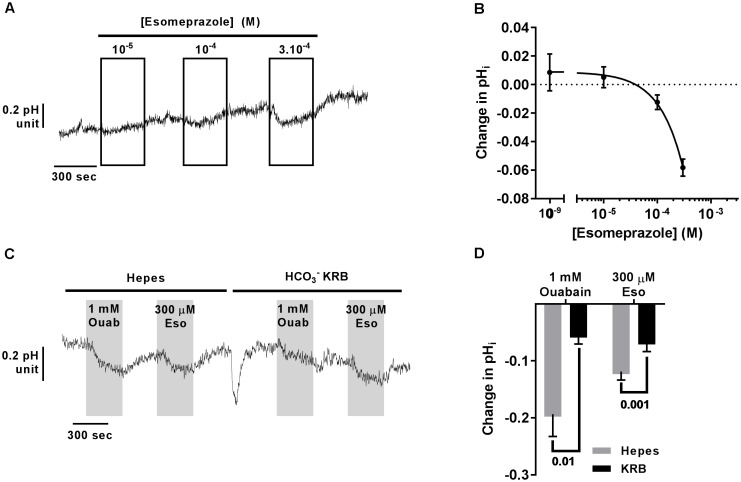
Acute esomeprazole decreases intracellular pH in primary CF hAECs by inhibiting H^+^ secretion. **(A)** Representative trace of the effect of increasing concentrations of acute esomeprazole on pH_i_. **(B)** Summary data of the esomeprazole dose response on pH_i_ (*n* ≥ 7, three donors). **(C)** Representative trace of the effect of 1 mM apical ouabain (Ouab) or 300 μM apical esomeprazole (Eso) in the absence (Hepes) or presence of ${\text{HCO}}_{3}^{-}$ (${\text{HCO}}_{3}^{-}$ KRB). **(D)** Summary data of the effect of 1 mM apical ouabain (Ouab) or 300 μM apical esomeprazole (Eso) in the absence (Hepes) or presence of ${\text{HCO}}_{3}^{-}$ (${\text{HCO}}_{3}^{-}$ KRB) presented as mean ± SEM (*n* = 3, two donors, two-way ANOVA with Sidak’s post-test).

Using Blastn, we found that the mRNA of *ATP12A*, the non-gastric H^+^/K^+^-ATPase, shares 70% identity with the gastric H^+^/K^+^-ATPase, ATP4A mRNA, which translates to around 65% identity in the protein products (Blastp). This H^+^ pump is targeted in gastric ulcers and gastroesophageal reflux diseases (GERDs) by antacids, and especially PPIs. We therefore investigated the effect of the PPI, esomeprazole (Eso) on pH homeostasis in CF hAECs. It has been previously published that airway epithelial cells do not express the gastric H^+^ pump and this was confirmed in our cells in which we found an average Ct value of 37.50 ± 1.53 in CF hAECs and 40 in non-CF hAECs (compared to averaged values of 21.61 ± 0.21 in CF cells and 22.03 ± 0.66 in non-CF cells for *ATP12A*, Supplementary Figure 4). We then assessed the effect of increasing concentrations of apical Eso on pH_i_ and showed that CF hAECs responded in a dose dependent manner (Figures 5A,B). To elucidate whether Eso targeted H^+^ or ${\text{HCO}}_{3}^{-}$ transport, pH_i_ experiments were performed in the absence (Hepes) or presence of ${\text{HCO}}_{3}^{-}$ (${\text{HCO}}_{3}^{-}$ KRB). Figures 5C,D show that Eso, as well as ouabain, inhibited H^+^ secretion and that the effect of both was enhanced in the absence of ${\text{HCO}}_{3}^{-}$.

**FIGURE 6:**
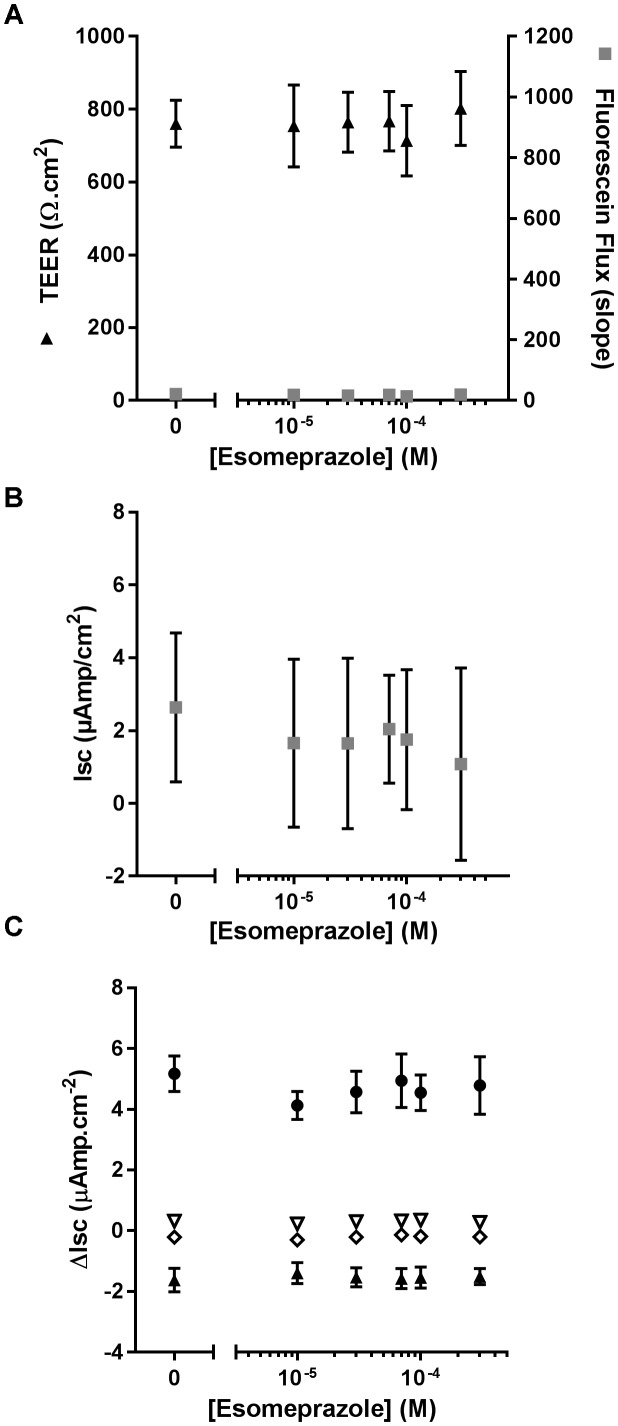
Chronic esomeprazole does not disrupt epithelial integrity of CF hAECs. **(A)** Effect of increasing concentrations of apical esomeprazole on TransEpithelial Electrical Resistance (TEER) and Fluorescein Flux measured in Ussing chambers in the presence of a basolateral to apical Cl^-^ gradient (*n* = 5, five donors). **(B,C)** Effect of increasing concentrations of apical eso on resting short-circuit current (Isc, **B**, *n* = 5), and **(C)** amiloride-sensitive Isc (ΔIsc_(amil)_, black triangle, *n* = 5), Fsk-induced Isc (ΔIsc_(Fsk)_, open triangle, *n* = 5), CFTRinh172-sensitive Isc (ΔIsc_(172)_, open diamond, *n* = 5) and UTP-induced Isc (ΔIsc_(UTP)_, black circles, *n* = 5).

As ouabain caused detrimental effects on epithelial integrity, we then evaluated the effect of chronic exposure of CF hAECs to increasing concentrations of Eso on TEER, fluorescein flux and ion channel activity. Concentrations of Eso, ranging from 10 to 300 μM, showed no effect on any of these parameters (Figures 6A–C) supporting a non-toxic effect of Eso on CF hAECs.

**FIGURE 7:**
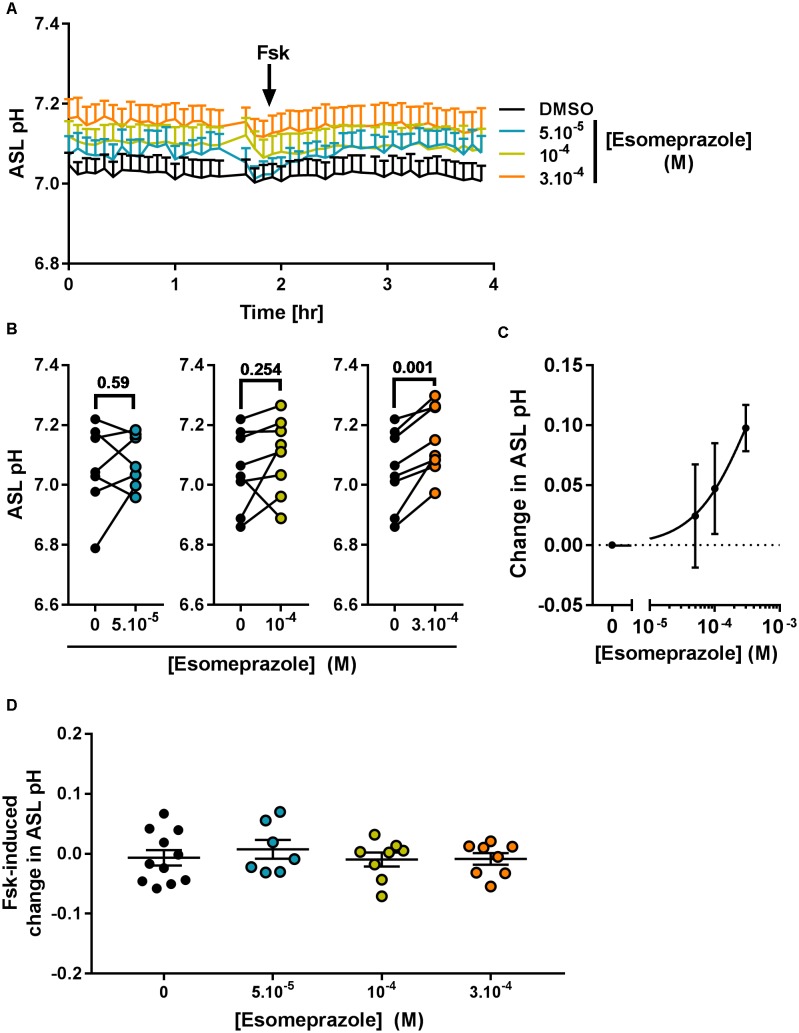
Chronic esomeprazole increases ASL pH in primary CF hAECs. CF hAECs were treated overnight with 50, 100, or 300 μM apical esomeprazole and ASL pH was measured under resting condition (**A–C**, *n* ≥ 7, four to six donors, paired *t*-test) and after stimulation with forskolin (**A,D**, Fsk, basolateral, 10 μM, *n* ≥ 7, four to six donors, ANOVA with Dunnett’s post-test). **(A)** Mean kinetic data of ASL pH of CF hAECs treated with increasing concentrations of esomeprazole. **(B)** Paired data of ASL pH for each independent experiment after treatment with 50 μM (left panel), 100 μM (middle panel), or 300 μM (right panel) of Eso. **(C)** Dose response of ASL pH in response to Eso. **(D)** Forskolin-induced changes in ASL pH in cells treated with 50, 100, 300 μM esomeprazole.

Considering its effect on pH_i_ (Figure 5) we then investigated the effect of Eso on ASL pH. Primary CF hAECs were treated overnight with 50, 100, or 300 μM Eso and the next day ASL pH was monitored for 2 h before FSK was added basolaterally. Although increasing concentrations of Eso appeared to increase ASL pH in a dose-dependent manner (Figure 7A) it only reached significance with the highest dose tested, 300 μM, which raised resting ASL pH by 0.10 ± 0.02 (*p* = 0.001, *n* = 8, paired *t*-test, Figures 7A–C) but did not change the response to Fsk by CF hAECs (Figure 7D). This result, taken together with the Ussing chamber experiments in which Eso did not change either Fsk-or UTP induced Isc (Figure 6C), suggests that Eso does not increase ASL pH in CF hAECs via an increase in either mutant CFTR, or calcium-mediated anion secretion, but rather, it works via inhibiting proton secretion.

### Chronic Esomeprazole-Induced Alkalization of the ASL but Not Acute Intracellular Acidification Is ATP12A Dependent

**FIGURE 8:**
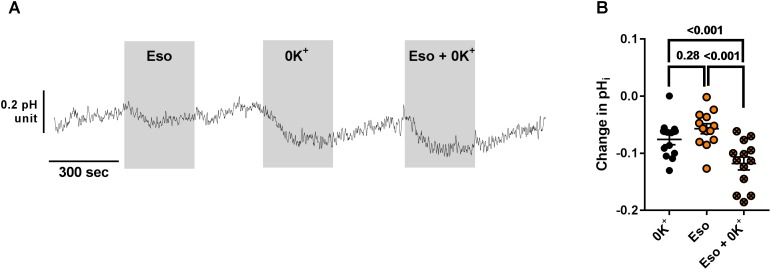
Additive effect of acute esomeprazole and K^+^-free solution on pH_i_. **(A)** CF hAECs were exposed acutely to apical esomeprazole (Eso, 300 μM), K^+^-free solution (0K^+^) or both. **(B)** Summary data of the effect of Eso and K^+^ free solution on pH_i_ (*n* = 11, four donors, Repeated Measures (RM)-ANOVA with Tukey post-test).

We then investigated the molecular target of acute and chronic exposure to Eso. Intracellular pH was monitored during exposure to acute Eso, 0K^+^, or Eso + 0K^+^. Eso and 0K^+^ induced intracellular acidification in a similar manner (Figure 8, *p* = 0.28, *n* = 11, RM ANOVA). However, Eso and 0K^+^ induced a further decrease in pH_i_ when compared to either 0K^+^ alone (Figure 8B, *n* = 13, *p* < 0.001, RM ANOVA) or to Eso alone (Figure 8B, *n* = 13, *p* < 0.001, RM ANOVA) suggesting that the acute effect of Eso on pH_i_ is independent of ATP12A and targets another H^+^ transporter. Similar results were found using ouabain (1 mM) instead of 0K^+^ in the same type of experiment (Supplementary Figure 5, ouabain vs. Eso+ouab *p* = 0.03; Eso vs. Eso+ouab *p* < 0.001; *n* = 10, RM ANOVA). Furthermore, using CF hAECs in which ATP12A expression was reduced by CRISPR-Cas9 (Supplementary Figure S6C), we found that 0K^+^ and ouabain-induced acidification were reduced by 44 and 52%, respectively (Supplementary Figures 6A,B) whereas Eso-induced acidification was only decreased by 9% (Supplementary Figures 6A,B).

**FIGURE 9:**
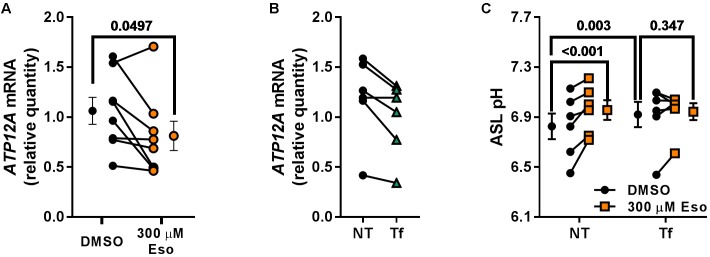
Chronic esomeprazole treatment targets ATP12A. **(A)** CF hAECs were treated for 24 h with 300 μM apical esomeprazole (Eso) and *ATP12A* mRNA levels measured by RT-qPCR (*n* = 8, four donors, paired *t*-test). **(B)** CF hAECs were transfected with the CRISPR-Cas9 vector, PX462, containing gRNA sequences targeting upstream and downstream of *ATP12A* exon 2. ATP12A knock-down was confirmed by RT-qPCR which showed a decrease of 17.1 ± 4.4% (NT: non-transfected, Tf: Transfected; *n* = 6, four donors, paired *t*-test). **(C)** CF hAECs transfected or not were treated overnight with 300 μM Eso and ASL pH was monitored the following day (*n* = 6, four donors, two-way ANOVA with Tukey post-test).

In contrast, we identified ATP12A as a potential target of chronic Eso treatment in CF hAECs. Indeed, RT-qPCR showed that 24 h treatment with 300 μM Eso decreased significantly mRNA levels of *ATP12A* (Figure 9A, *p* = 0.049, *n* = 7, paired *t*-test). We confirmed this result using the CRISPR-Cas9 method to knock down *ATP12A* expression. Results showed that in cells where *ATP12A* levels were reduced (Figure 9B, -17.1 ± 4.4%, *p* = 0.02, *n* = 6, paired *t*-test), ASL pH increased (Figure 9C, *p* = 0.003, *n* = 6, two-way ANOVA) and chronic Eso was not able to induce a further increase in ASL pH (Figure 9C, *p* = 0.347, *n* = 6, two-way ANOVA).

## Discussion

In the past few years, there has been increasing evidence that extracellular ASL pH plays a major role in airway homeostasis and defense against inhaled pathogens. Thus an acidic pH was shown to reduce bacterial killing (Pezzulo et al., 2012), increase ENaC activity and therefore ASL dehydration (Garland et al., 2013; Tan et al., 2014) and increase mucus viscosity (Tang et al., 2016). An acidic ASL in CF airways can be explained by a defect in ${\text{HCO}}_{3}^{-}$ secretion (due to defective CFTR) and/or an unregulated H^+^ secretion. Recently, it was shown that ATP12A, was responsible for this unchecked H^+^ secretion in human and pig CF airways and accounted for the increased mucus viscosity and decreased bacterial killing ability (Shah et al., 2016) promoting this pump as a valid target for CF airway disease.

We have shown that inhibition of the non-gastric H^+^/K^+^-ATPase using ouabain concentrations close to the IC_50_ significantly increased ASL pH in CF hAECs. However, this increase in ASL pH was not stable after overnight incubation with the drug and a slow, but constant, alkalinization was observed throughout the ASL pH measurement. This led us to suggest that upon chronic treatment with apical ouabain, the epithelia were becoming leaky, allowing for the equilibration of the pH between the basolateral (pH 7.4) and the apical compartments. In kidney epithelia, it has been shown that chronic treatment (3 days) with a low dose (1 μM) of ouabain induced the disassembly of tight-junction proteins as well as cell adhesion associated protein, leading to cell to cell and cell to surface detachment (Larre et al., 2010; Rincon-Heredia et al., 2014). Using a shorter treatment time frame, we showed that ouabain disrupted CF airway epithelial integrity in a dose-dependent manner, leading to an increase in LDH release, fluorescein flux as well as a profound drop in transepithelial resistance. It was also linked to a decrease in ENaC-mediated and calcium-activated transepithelial transport, but not to an increase in CFTR conductance (data not shown). The lack of effect of ouabain on CFTR in CF cells is in contrast to the study from Zhang et al. (2012) which showed that 24 h treatment with low concentrations of cardiac glycosides, including ouabain, induced trafficking of the F508del-CFTR to the cell surface in a cell line over-expressing the mutated CFTR, mimicking the effect of low temperature incubation (Zhang et al., 2012). This may be explained either by the difference in concentrations used (0.1 vs. 10–300 μM) or by a difference in the cell models. Indeed, the cell line used in Zhang’s study overexpressed F508del-CFTR and it is therefore more likely to detect small changes in CFTR trafficking and/or transport activities and report effects that do not happen in a non-overexpressing systems. Our experiments were performed on primary CF hAECs from three different donors (all F508del homozygous). Inter-donor variability was compensated for by doing the statistical analysis using paired/repeated measure settings and therefore we are confident about the lack of effect of ouabain on mutant CFTR activity.

Surprisingly and contrary to what other groups have published (Shah et al., 2016), forskolin did not appear to stimulate ATP12A and acidify the ASL (Figures 2A, 7A,D). We confirmed our results, by measuring the activity of ATP12A (using apical 0K^+^ or ouabain) in the presence or absence of forskolin. The results showed that (i) forskolin did not induce any change in pH_i_ (Supplementary Figure 2) and (ii) the ouabain or 0K^+^-induced changes in pH_i_ were not significantly different whether forskolin was present or not, indicating that ATP12A activity was not altered by a rise in cAMP. The study by Shah et al. (2016) showed that Fsk and IBMX induced CF ASL acidification, but did not explicitly demonstrate the role of ATP12A in this process in *human* cells. Moreover, in cultured porcine airway epithelial cells, ouabain only partially inhibited the cAMP-induced acidification. Although we cannot ascertain the reason behind this discrepancy, differences in the methodology in assessing ASL pH or culture conditions could potentially explain it.

Several groups have studied the *in vivo* effect of cardiac glycosides, including ouabain, in inhaled form, on bronchial reactivity in asthma (Agrawal et al., 1986; Knox et al., 1988; Hulks and Patel, 1989). Indeed in the 1980s, a study conducted in England and Wales showed a correlation between asthma mortality and dietary salt intake (Burney, 1987) and it was thought that inhibiting Na^+^/K^+^-ATPase would increase the intracellular concentration of Na^+^, inhibiting Na^+^-Ca^2+^ exchange, and thereby increasing contractility of smooth muscle cells. In one study, Agrawal et al. (1986) showed that a low dose of ouabain-induced bronchodilatation in 6 out of 10 asthmatic subjects, whereas higher doses induced bronchoconstriction. However, two more studies reported no effect of inhaled ouabain on FEV_1_ or bronchial reactivity to histamine in asthmatic patients (Knox et al., 1988; Hulks and Patel, 1989). Only one study has reported the effect of topical ouabain in the airways of CF patients (originally to study the role of the Na^+^/K^+^-ATPase on Nasal Potential Difference (NPD) in CF nasal epithelium) and showed no effect on NPD in people with CF and control subjects (Peckham et al., 1995). However, this was done over a short period of time: NPD was measured every 15 min for an hour after application of ouabain. This time-frame would be too short to observe any effect on pH. Taken together with our results, these studies suggest that ouabain is not a good candidate to target pH homeostasis as a means to improve CF airway disease.

Because of the significant sequence homology between different members of the proton/potassium ATPase family, and the fact that PPIs inhibit the gastric ATPase by binding to cysteine residues, we hypothesized that these inhibitors could also target the non-gastric H^+^/K^+^-ATPase. To test this we investigated the effect of the PPI esomeprazole. It belongs to the second generation of PPIs, which are more stable than the 1^st^ generation, and remain bound to the gastric H^+^/K^+^-ATPase, thus inhibiting H^+^ secretion until new proteins are synthesized (Andersson et al., 2001). Importantly, our results showed that esomeprazole increased the ASL pH in CF hAECs, and furthermore, that this was not accompanied by any deleterious effects as observed with ouabain treatment. Although the intracellular acidification induced by acute esomeprazole exposure appeared to be independent of the non-gastric H^+^/K^+^-ATPase, its chronic effect on ASL pH was linked to a decrease in *ATP12A* mRNA expression (Figure 9A) and CRISPR-Cas9 experiments suggested that esomeprazole targeted the non-gastric H^+^/K^+^-ATPase in order to increase ASL pH (Figure 9B,C). Interestingly, the inhibitory effect of PPIs on ATP12A has been reported as a potential therapeutic approach in treating the inflammatory process in chronic rhinosinusitis (Min et al., 2017). PPIs are mainly prescribed for GERD and the association between GERD and lung diseases has been established although the mechanism of action and causality have not. Accordingly, there has been an interest in the effect of PPI on chronic lung diseases and although results are controversial (Laheij et al., 2004; Filion et al., 2014; Benson et al., 2015; Lee et al., 2015; Othman et al., 2016), PPIs appear to have anti-inflammatory properties in COPD and IPF (Ghebremariam et al., 2015; Xiong et al., 2016). One of the hallmarks of the lung pathophysiology in CF is the chronic inflammation. It is characterized by an excessive neutrophilic infiltration of the airways, abundant protease (neutrophil elastase (Le Gars et al., 2013) and bacterial elastase (Saint-Criq et al., 2018)) and elevated levels of IL-1 and IL-8 in the CF airways lumen (Konstan et al., 1994; Balough et al., 1995; Armstrong et al., 1997; Montgomery et al., 2018). We can therefore hypothesize that the use of esomeprazole in CF could also dampen the chronic inflammation observed in CF airways. In addition to anti-inflammatory properties, PPIs have shown antioxidant features in various cell types, another hallmark of the CF airway pathophysiology mainly due to the large neutrophilic infiltration. *In vitro*, the first generation PPI, omeprazole, was shown to scavenge hypochlorous acid (Lapenna et al., 1996) and *in vivo*, this PPI prevented stress-induced ulcer formation by blocking the generation of ^∗^OH (Biswas et al., 2003), whereas esomeprazole prevented the depletion of the antioxidant glutathione induced by indomethacin in rat gastric mucosa (Pastoris et al., 2008). PPIs have also shown antimicrobial properties. *In vitro*, esomeprazole decreased *Pseudomonas aeruginosa* and *Staphylococcus aureus* biomass and increased killing by conventional antibiotics. It also largely inhibited biofilm formation by these two bacterial species (Singh et al., 2012). Sasaki also reported an antiviral effect of lansoprazole in tracheal cells by reducing their susceptibility to rhinovirus infection (through a decrease in ICAM-1 that serves as the receptor for rhinovirus) (Sasaki et al., 2005). Although we have not determined the effects of chronic esomeprazole on ASL hydration, mucus viscosity or bacterial killing in primary CF hAECs, other groups have reported that small changes in ASL pH (<0.2 pH) were linked to an increased bactericidal activity and a decreased mucus viscosity in human non-CF airway cells (Shah et al., 2016). This suggests that the changes in ASL pH we have observed could have beneficial effects in CF airway disease.

In CF, 80 to 90% of patients have silent or symptomatic gastroesophageal reflux (Ledson et al., 1998; Button et al., 2005; Blondeau et al., 2008) and therefore a large proportion of these patients use PPIs. PPIs serve to reduce acid reflux but also compensate for the lack of gastrointestinal secretion (due to defective CFTR), and thereby enhance the benefit of pancreatic enzyme replacement therapy. Indeed, several studies have shown the beneficial effect of PPI use in children with CF when looking at steatorrhea and nutritional status (Tran et al., 1998; Proesmans and De Boeck, 2003), and it is well acknowledged that in these cases, PPIs such as omeprazole and lansoprazole, show improvement in fat absorption. However, the effect of PPI on pulmonary function is more controversial. PPIs decrease acid content of gastric juice and Palm et al. (2012) showed that there was a negative correlation between non-acid reflux burden and FEV_1_. Another study, looking at children with CF between 2009 and 2014, reported that PPI use was associated with a decline in percent predicted (pp)FEV_1_ as well as an increase in future pulmonary exacerbation rate (van Horck et al., 2018). This was also reported in adults with CF in which PPI use was associated with an increase in the number of hospitalizations due to pulmonary exacerbations (Ayoub et al., 2017). However, by differentiating those on gastric acid inhibitors because of GERD, from those taking PPIs because of fat malabsorption, van der Doef et al. (2009) showed that only the children with CF taking gastric acid inhibitors because of GERD presented a reduced pulmonary function. This challenges the role of PPIs in pulmonary exacerbations in CF and suggests that GERD rather than PPI use is involved in pulmonary decline.

Since our current study showed a beneficial effect of esomeprazole on CF airway ASL pH homeostasis, and considering the other potential benefits of PPIs against bacterial and viral infections, oxidative stress and inflammatory processes, we propose that inhaled use of PPIs in CF airways might alleviate CF lung pathophysiology. Interestingly, novel H^+^/K^+^-ATPase inhibitors, termed P-CABs (Potassium-Competitive Acid Blockers), such as Vonoprazan, are more potent than PPIs for eradication of clarithromycin-resistant *Helicobacter pylori* (Li et al., 2018). Thus it will be interesting to test the effects of these new H^+^ pump inhibitors on inflammation, infection, and pH homeostasis in CF airways.

## Ethics Statement

The cells were obtained under protocol #03-1396 approved by the University of North Carolina at Chapel Hill Biomedical Institutional Review Board.

## Author Contributions

LD, JT, AY, and SR performed experiments and analyzed data. MG designed and performed experiments, provided reagents, analyzed data and edited the manuscript. VS-C designed and performed experiments, analyzed data and wrote the manuscript.

## Conflict of Interest Statement

The authors declare that the research was conducted in the absence of any commercial or financial relationships that could be construed as a potential conflict of interest.

## Footnotes

1 crispr.mit.edu/
