# Supplementary material for: Esomeprazole Increases Airway Surface Liquid pH in Primary Cystic Fibrosis Epithelial Cells
Source: Front Pharmacol. 2018 Dec 11;9:1462. doi: 10.3389/fphar.2018.01462 (PMC6297391; doi:10.3389/fphar.2018.01462)
Supplement: Supplementary file 1 [file Presentation_1.pdf]

Supplementary Material

Esomeprazole increases airway surface liquid pH in primary Cystic Fibrosis epithelial cells

Livia Delpiano, Joseph J. Thomas, Annabel R. Yates, Sarah J. Rice, Michael A. Gray, Vinciane Saint-Criq*

* **Correspondence:** Corresponding Author: vinciane.saint-criq@newcastle.ac.uk

1 Supplementary Figures

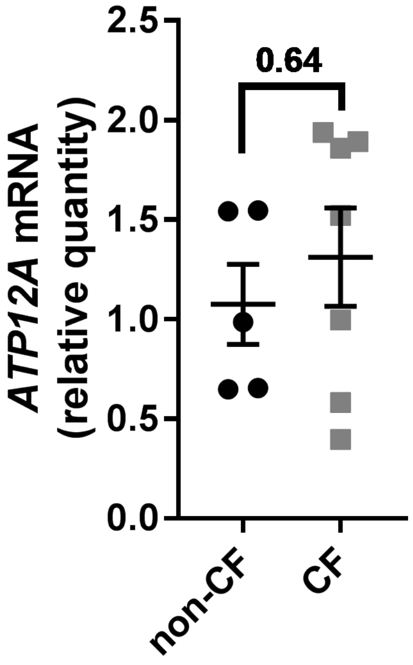

Supplementary Figure 1: The non-gastric H^+/K^+-ATPase is expressed at the same level in non-CF and CF primary hAECs. Relative mRNA quantity of *ATP12A* in non-CF (black circles, n=5, three donors) and CF (grey squares, n=7, three donors) hAECs (Mann-Whitney test).

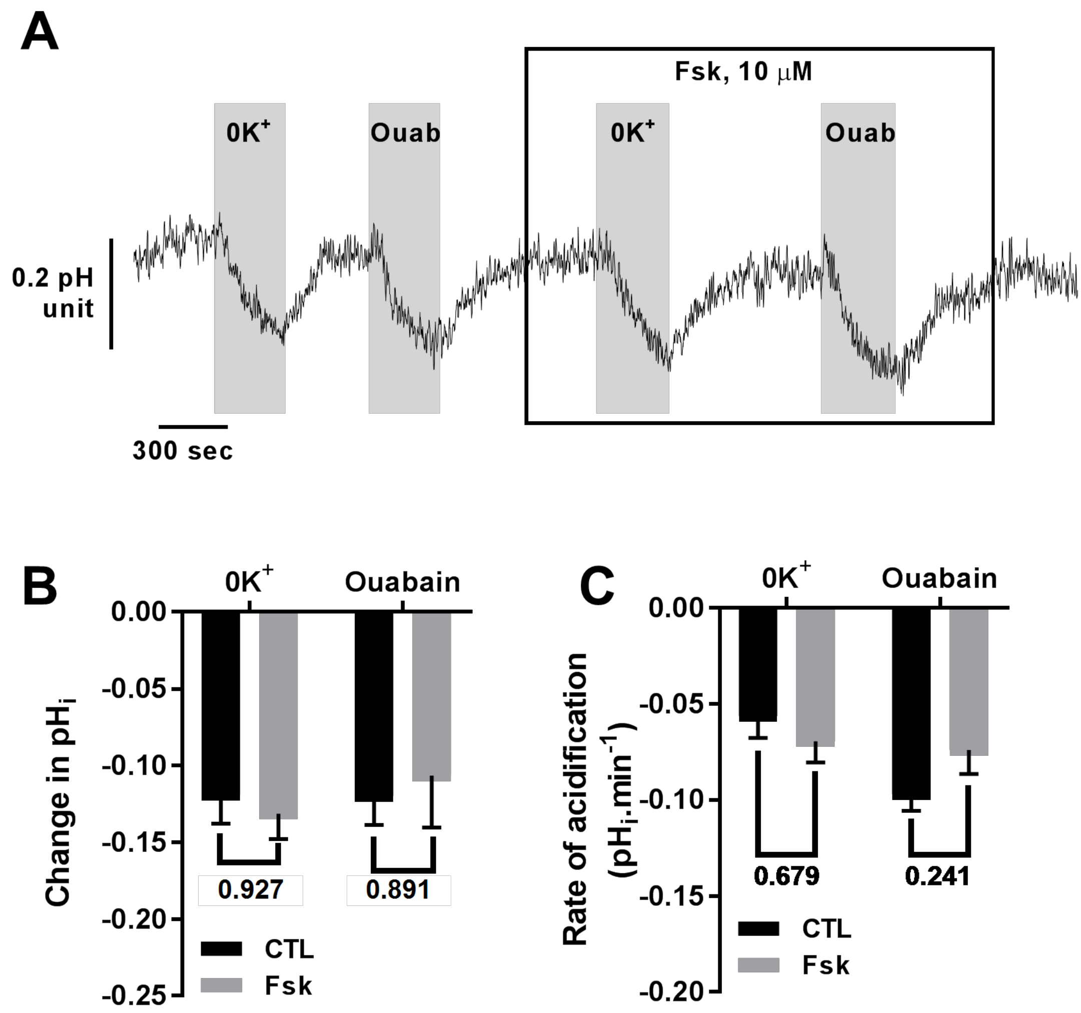

Supplementary Figure 2: Forskolin does not affect $0K^+$ and ouabain-induced acidification. (A) Representative trace of acute apical effect of $0K^+$ and ouabain (Ouab) in the absence or presence of Forskolin (Fsk, 10 μM) on pH_i. (B) Summary data of the effect of Fsk on $0K^+$ and ouabain-induced changes in pH_i ($n \geq 5$, two donors, $p>0.05$, two-way ANOVA with Sidak's post-test). (C) Summary data of the effect of Fsk on the rate of acidification induced by $0K^+$ and ouabain ($n \geq 5$, two donors, $p>0.05$, two-way ANOVA with Sidak's post-test).

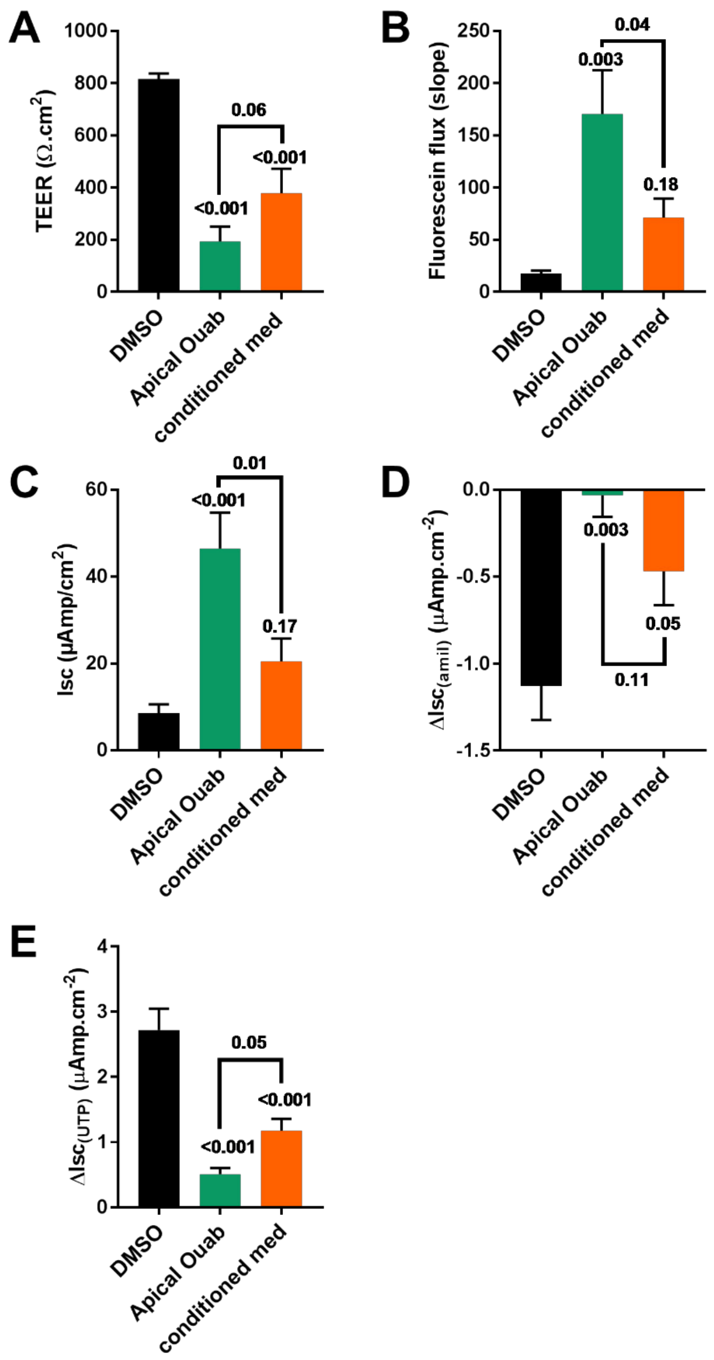

Supplementary Figure 3: Basolateral treatment of CF hAECs with conditioned medium from cells treated with 70 µM apical ouabain partly reproduced the deleterious effect of apical ouabain treatment. Effect of apical ouabain (Ouab) and conditioned medium (conditioned med.) on TransEpithelial Electrical Resistance (TEER, A) and Fluorescein Flux (B) assessed in Ussing chambers in the presence of a basolateral to apical Cl^- gradient (n=6, two donors, one-way ANOVA, Holm-Sidak correction). Effect of apical ouabain and conditioned medium on resting short-circuit current (Isc, C, n=6), amiloride-sensitive Isc ($\Delta Isc_{(amil)}$, D, n=6) and UTP-induced Isc ($\Delta Isc_{(UTP)}$, E, n=6).

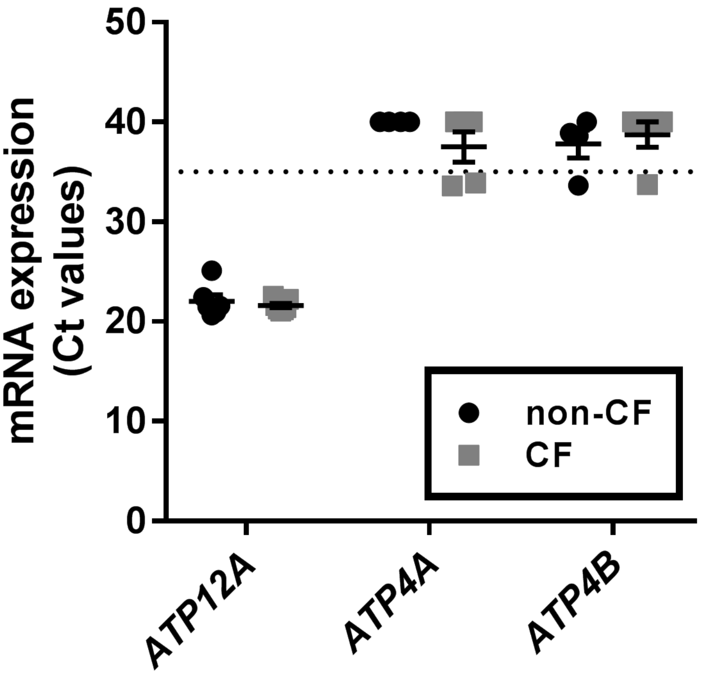

Supplementary Figure 4: The gastric H^+/K^+-ATPase is not expressed in non-CF and CF primary hAECs. Threshold cycle (Ct) values for ATP12A (n=5 non-CF, three donors, n=7 CF, three donors), ATP4A and ATP4B genes (n=4 non-CF, three donors, n=5 CF, three donors).

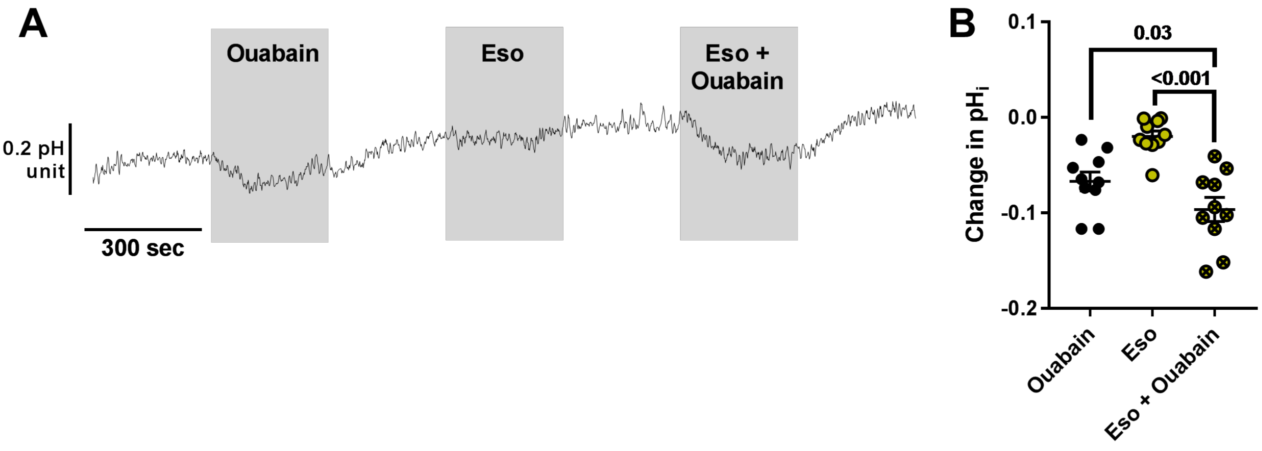

Supplementary Figure 5: Additive effect of acute esomeprazole and ouabain on pH_i. (A) Representative trace of the effect of acute apical ouabain alone (1 mM), esomeprazole alone (Eso, 100 μM) or a combination of both on intracellular pH (pH_i). (B) Summary data of the effect of ouabain alone, esomeprazole alone or a combination of both on pH_i (n=10, four donors; repeated measures (RM)-one-way ANOVA).

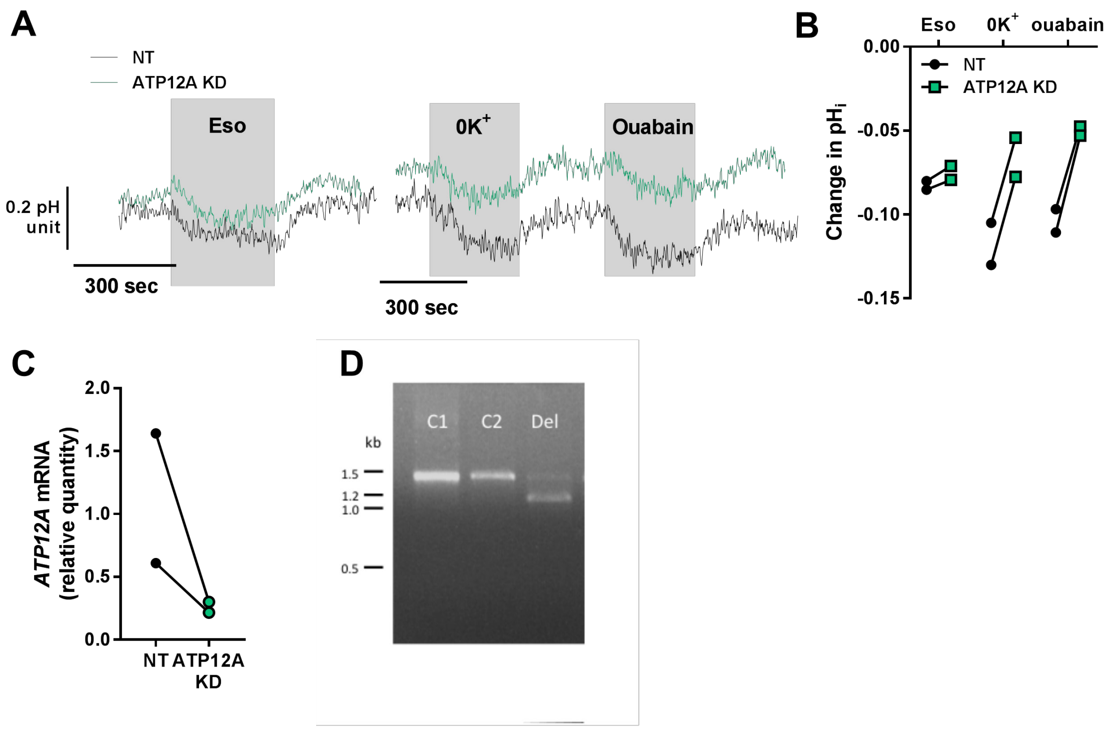

Supplementary Figure 6: Acute esomeprazole does not target ATP12A. (A-B) Traces (A) and summary data (B, n = two donors) of pH_i measurements in response to esomeprazole (Eso, apical, 300µM), K^+-free solution ($0K^+$, apical) or ouabain (apical, 1 mM) in cells knocked-down for *ATP12A*. (C) *ATP12A* mRNA levels in non-transfected CF hAECs (NT) and *ATP12A* CRISPR-Cas9 edited cells (ATP12A KD). (D) Confirmation of the deletion of *ATP12A* exon 2 on agarose gel showing PCR analyses from purified genomic DNA of C1= blood, C2= human chondrocyte cell line Tc28a2 DNA (non-targeting cas9), Del= Tc28a2 expressing gRNAs targeting *ATP12A* exon 2 showing the deletion (390bp).
